# Differential Expression of DeltaFosB in Reward Processing Regions Between Binge Eating Prone and Resistant Female Rats

**DOI:** 10.3389/fnsys.2020.562154

**Published:** 2020-10-16

**Authors:** Richard Quansah Amissah, Sandrine Chometton, Juliane Calvez, Genevieve Guèvremont, Elena Timofeeva, Igor Timofeev

**Affiliations:** ^1^Faculté de Médecine, Département de Psychiatrie et de Neurosciences, Centre de Recherche de L’Institut Universitaire de Cardiologie et de Pneumologie de Québec, Université Laval, Québec, QC, Canada; ^2^Faculté de Médecine, Département de Psychiatrie et de Neurosciences, Centre de Recherche du CERVO, Université Laval, Québec, QC, Canada

**Keywords:** deltaFosB immunoreactivity, reward, compulsivity, binge eating prone, binge eating resistant, foot-shock stress

## Abstract

Binge eating (BE) is characterized by the consumption of large amounts of palatable food in a discrete period and compulsivity. Even though BE is a common symptom in bulimia nervosa (BN), binge eating disorder (BED), and some cases of other specified feeding or eating disorders, little is known about its pathophysiology. We aimed to identify brain regions and neuron subtypes implicated in the development of binge-like eating in a female rat model. We separated rats into binge eating prone (BEP) and binge eating resistant (BER) phenotypes based on the amount of sucrose they consumed following foot-shock stress. We quantified deltaFosB (ΔFosB) expression, a stably expressed Fos family member, in different brain regions involved in reward, taste, or stress processing, to assess their involvement in the development of the phenotype. The number of ΔFosB-expressing neurons was: (1) higher in BEP than BER rats in reward processing areas [medial prefrontal cortex (mPFC), nucleus accumbens (Acb), and ventral tegmental area (VTA)]; (2) similar in taste processing areas [insular cortex, IC and parabrachial nucleus (PBN)]; and (3) higher in the paraventricular nucleus of BEP than BER rats, but not different in the locus coeruleus (LC), which are stress processing structures. To study subtypes of ΔFosB-expressing neurons in the reward system, we performed *in situ* hybridization for glutamate decarboxylase 65 and tyrosine hydroxylase (TH) mRNA after ΔFosB immunohistochemistry. In the mPFC and Acb, the proportions of γ-aminobutyric acidergic (GABAergic) and non-GABAergic ΔFosB-expressing neurons were similar in BER and BEP rats. In the VTA, while the proportion of dopaminergic ΔFosB-expressing neurons was similar in both phenotypes, the proportion of GABAergic ΔFosB-expressing neurons was higher in BER than BEP rats. Our results suggest that reward processing brain regions, particularly the VTA, are important for the development of binge-like eating.

## Introduction

Eating disorders, namely anorexia nervosa (AN), bulimia nervosa (BN), binge eating disorder (BED), and other specified feeding or eating disorders (OSFEDs), cause severe disturbances to eating habits (Galmiche et al., 2019). Hudson et al. (2007) reported a lifetime prevalence rate of 0.6% for AN (0.9% of women and 0.3% of men), 1% for BN (1.5% of women and 0.5% of men), and 3% for BED (3.5% of women and 2.0% of men), which suggests that females are more prone to eating disorders than males (Kessler et al., 2013). Binge eating (BE) is a core symptom in BN, BED, and some cases of OSFEDs and affects about 4.5% of the general population (Hudson et al., 2007); however, its underlying mechanisms are still poorly understood (Sinclair et al., 2015).

BE is characterized by eating a large amount of palatable food than would normally be consumed in a discrete amount of time and a loss of sense of control during the bingeing episode (American Psychiatric Association, 2013). In humans, binge eating is triggered by factors that may be neurological (changes in neurotransmitters), psychological (anger, depression, stress), societal, or interpersonal (Hetherington, 2000). While several neuroimaging studies, using functional magnetic resonance imaging (fMRI) in humans, showed that BE is associated with increased fMRI activity in the reward system (Karhunen et al., 2000; Schafer et al., 2010; Filbey et al., 2012; Tanofsky-Kraff et al., 2013; Lee et al., 2017), others reported decreased fMRI activity in this system (Balodis et al., 2013, 2014; Halpern et al., 2013; Reiter et al., 2017). It is therefore unclear whether BE is associated with increased or decreased reward system activity and whether other systems could be involved. This important knowledge will help develop efficient therapies that could target specific neuron populations in the different brain regions that form the reward system.

To study BE, rodent models developed using intermittent access to palatable food and either food restriction or stress, or both were proposed (Corwin and Babbs, 2012). One of the models is based on the consumption of palatable foods, and assigned rats into two groups [binge eating prone (BEP) or binge eating resistant (BER)] based on their 4-h intake (Boggiano et al., 2007). However, the contribution of stress to binge-eating behavior is important in humans (Harrington et al., 2006), and females are more prone to eating disorders than males (Kessler et al., 2013). Our laboratory recently developed a modified binge-like eating female rat model using intermittent access to sucrose solution and foot-shock stress without food restriction that mimics several clinical features of BE (Calvez and Timofeeva, 2016), resulting in binge eating prone (BEP; ≈30% of rats) and binge eating resistant (BER; ≈30% of rats) rat phenotypes.

Several studies have been conducted which investigated reward system activity in rats. The study by Sinclair et al. (2015) reported increases in activity in the nucleus accumbens (Acb) and medial prefrontal cortex (mPFC) of bingeing rats based on the number of c-fos-expressing cells in these regions, in response to palatable food consumption. C-fos expression in response to the reward-cue presentation was reported in both the Acb and the dorsal striatum of adult rats and during pubertal development (Friemel et al., 2010) while Wallace et al. (2008) and Muñoz-Escobar et al. (2019) showed that deltaFosB (ΔFosB) is expressed in the mPFC and Acb following repeated consumption of palatable food. These studies show that reward system activity is important for the consumption of palatable food and it is altered in binge-like eating rats. However, the involvement of this system during the development of the binge-eating phenotype is not well known.

We hypothesized that the reward system, as well as taste- and stress-mediating brain regions, are involved in the development of binge-like eating. Thus, the goal of the study was to identify brain regions implicated in the development of binge-like eating, possible differences in neuronal activity between BEP and BER rats in these regions, and the neuron types implicated in these regions. Most studies used the c-fos expression to evaluate the effect of acute neuronal stimulation by palatable food consumption in binge-like eating rodents (Bello et al., 2009; Sinclair et al., 2015). However, c-fos is transiently expressed and degrades rapidly (Herrera and Robertson, 1996). We, therefore, opted for ΔFosB because it persists for long periods due to its high stability (Nestler et al., 2001). ΔFosB accumulates in neurons after chronic stress (Perrotti et al., 2004), chronic treatment with drugs (Cunningham et al., 2008; Perrotti et al., 2008), and chronic sucrose consumption (Wallace et al., 2008). Since our binge-like eating model reproduces the consumption of palatable food and is triggered by stress, we aimed to analyze ΔFosB expression in brain regions which process reward [mPFC, Acb, and ventral tegmental area (VTA; Richard et al., 2013a)], taste [parabrachial nucleus (PBN) and IC (Lundy and Norgren, 2004)], and stress [paraventricular hypothalamic nucleus (PVN) and locus coeruleus (LC; Ziegler et al., 1999)]. The results showed that the brain regions implicated in the development of binge-like eating are mainly in the reward system. In this system, the VTA may play a fundamental role.

## Materials and Methods

All experiments were performed following the guidelines of the Canadian Council on Animal Care and approved by the Université Laval Committee on Ethics and Animal Research (protocol 2017013).

### Animals

Forty naïve 45-day-old female Sprague–Dawley rats (body weight: 151–175 g) were purchased from the Canadian Breeding Laboratories (St-Constant, QC, Canada) for this study. Each rat was housed individually and maintained on a 12-h light/dark cycle with the dark cycle starting at 14:00 h in a housing facility with an ambient temperature of 23 ± 1°C. Unless otherwise stated, all rats had *ad libitum* access to tap water and standard rat chow (2018 Teklad Global 185 Protein Diet; 3.1 kcal/g, Harlan Teklad, Montreal, QC, Canada). We allowed 7 days for acclimatization of rats to the environmental conditions followed by 24-h access to 10% sucrose solution, 1 week before the start of experiments, to prevent neophobia to the taste of sucrose solution.

### Generation of Binge-Like Eating Rat Phenotypes

We generated the binge-like eating rat phenotype as described in a previous study (Calvez and Timofeeva, 2016). The protocol for generating the binge-like eating rat phenotype comprised of five non-stress sessions, followed by two stress sessions, a non-stress session, and a stress session (six non-stress sessions and three stress sessions in total). During each non-stress session, the rats were given 1-h *ad libitum* access to 10% sucrose solution (non-stress session), just at the start of the dark phase. The first non-stress session occurred on postnatal day (PD) 65. The interval between any two non-stress sessions was at least 2 days. Each stress session consisted of four foot-shocks with a direct current of 0.6 mA, lasting for 3 s, followed by 1-h access to 10% sucrose solution. The equipment for delivering the foot-shock comprises a chamber with a metal grid floor through which electrical current was sent. The inter-shock interval was 15 s. Consecutive stress sessions were separated by at least 3 days. Since this is a stress-induced binge-like eating model, only the consumption of sucrose during the three stress sessions was used to classify rats as either BEP or BER. For each stress session, the sucrose intake of all rats was ordered from highest to lowest and divided into tertiles. Any rat which appeared at least twice in the upper tertile and never in the lower tertile was considered BEP, while BER rats were rats which appeared at least twice in the lower tertile and never in the upper tertile (Calvez and Timofeeva, 2016). In this model, the proportions of rats identified to be BEP, BER, and intermediate (rats considered neither BEP nor BER) are approximately 30%, 30%, and 40%, respectively. In this study, 11 BEP and 12 BER female rats were obtained. They were subsequently divided into two cohorts (*n* = 6 and 5 for BEP, and *n* = 6/cohort for BER).

### Test for Compulsivity

A modified light/dark box was used to test for compulsivity in the first cohort. This test was conducted on PD80 according to a previously published study (Calvez and Timofeeva, 2016). It consists of a dark zone and a light zone. The light zone comprises a 30 cm × 30 cm box made of white Plexiglas while the black zone comprises a 30 cm × 30 cm box made of black Plexiglas. These two zones are connected by a 10-cm wide-open door. The light zone was brightly illuminated with a light of 300 lx considered aversive to rats (Kaplan et al., 1965). The dark zone was covered with a lid to allow a minimum amount of light as possible to enter (<5 lx). In the light zone, rats had free access to a 10% sucrose solution in a pre-weighed bottle. The experiment was conducted during the dark phase. Rats were first placed in the light compartment facing the spout of the sucrose bottle. The duration of the test session was 10 min. To distinguish the activity of rats around the sucrose bottle from activity elsewhere in the light zone, a demarcation (14 cm × 8 cm) around the sucrose bottle, called the zone of sucrose, was made. Rats which, despite the obvious aversive light condition, consumed high amounts of sucrose were considered compulsive (Dalley et al., 2011). The sucrose bottle was weighed before and after the 10 min-experiment to determine the quantity of sucrose consumed.

### DeltaFosB Immunohistochemistry

Three to four days after last access to the sucrose solution, rats were anesthetized using ketamine (160 mg/kg) and xylazine (20 mg/kg). After confirming that rats had no reflex upon pinching, they were intracardially perfused with 100 ml of ice-cold isotonic saline followed by 200 ml of 4% paraformaldehyde (PFA) solution. The rat brains were kept in 4% PFA at 4°C for 1 week. They were then transferred into 20% sucrose in 4% PFA overnight. Using a sliding microtome (Histoslide 2000, Heidelberger, Germany), we cut 30-μm thick coronal sections of brains and kept them at –30°C in a sterile cryoprotecting solution made of sodium phosphate buffer (50 mM), ethylene glycol (30%), and glycerol (20%) until they were processed for immunohistochemistry.

The primary antibody used for ΔFosB immunohistochemistry in this study stains both FosB and ΔFosB, but since FosB is known to degrade with time leaving the shorter 37 kD ΔFosB isoform after chronic stimulation (Nestler, 2004), we can confidently say that only a minority of the detected staining were contributed by FosB, similar to the antibody used in other studies (Cunningham et al., 2008, 2012).

Brain sections were first washed in 1% potassium phosphate-buffered saline (PPBS) solution followed by treatment with 30% H_2_O_2_ diluted in methanol (1:10). They were washed again in 1% PPBS and blocked for 1 h in a solution comprising 0.4% Triton-X, 2% bovine serum albumin, and 1% PPBS. The sections were incubated overnight at 4°C in the primary rabbit anti-ΔFosB antibody diluted in the blocking solution (sc-48; Santa Cruz Biotechnology, Santa Cruz, CA, USA, 1:1,000). The next day, sections were rinsed in 1% PPBS solution, followed by incubation for 1 h at room temperature in 1:1,500 biotinylated goat anti-rabbit immunoglobulin (Vector Laboratories Inc., Burlingame, CA, USA) diluted in blocking solution. The sections were then rinsed and transferred into a complex of horseradish peroxidase (HRP)-avidin solution (Vector Laboratories Inc., Burlingame, CA, USA) for 1 h at room temperature. It was washed with 1% PPBS and then with a tris-imidazole solution. To detect staining, a solution containing tris-imidazole, diaminobenzidine (DAB; 0.12 mg/ml), and 0.3% H_2_O_2_ was used. The sections were kept in DAB solution for 10 min, rinsed with PPBS, mounted on slides, and cover-slipped with DPX mounting medium.

### Double-Labeling for Neuron Subtypes

To study neurochemical subtypes of neurons that express ΔFosB, we used a glutamic acid decarboxylase 65 (GAD65) probe to identify GABAergic neurons and a tyrosine hydroxylase (TH) probe to identify dopaminergic neurons in brains of the second rat cohort. *In situ* hybridization was performed as described previously (Mitra et al., 2011). Following ΔFosB immunohistochemistry, sections were mounted on poly L-lysine coated slides and left to dry overnight under vacuum. The sections were subsequently fixed in 4% PFA for 20 min, exposed to proteinase K [10 μg/ml in 100 mM Tris-HCl containing 50 mM ethylenediaminetetraacetic acid (EDTA), pH 8.0] for 25 min to break down contaminating proteins, acetylated with acetylate anhydride (0.25% in 0.1 M triethanolamine, pH 8.0), and dehydrated by exposure to ethanol solutions of increasing concentration (50, 70, 95, and 100%). Afterward, the slides were vacuum dried for at least 2 h, followed by the addition of 90 μl of the hybridization solution to the slides. This solution contains an antisense ^35^S-labeled cRNA probe against GAD65 or TH. Coverslips were placed on the slides followed by overnight incubation at 55°C. After removal of coverslips the following day, the slides were washed in standard saline citrate (SSC; 0.6 M, 60 mM trisodium citrate buffer, pH 7.0), and exposed for 30 min to RNase-A at 37°C (20 μg/ml in 10 mM Tris-500 mM NaCl containing EDTA). They were then washed in decreasing concentrations of SSC (2×, 10 min; 1×, 5 min; 0.5×, 10 min; 0.1×, 30 min at 60°C), followed by dehydration in graded concentrations of ethanol. After vacuum drying for 2 h, the slides were defatted in xylene and later dipped in NTB2 nuclear emulsion. The slides were exposed for 7 days and then developed in D19 developer for 3.5 min at 14–15°C. They were later fixed in a rapid fixer (Eastman Kodak, Rochester, NY, USA) for 5 min. The slides were then washed for 1 h under running water, followed by counterstaining with Thionin (0.25%) and dehydration in graded concentrations of ethanol. They were cleared in xylene and cover-slipped following the application of DPX mounting medium.

To maintain RNA integrity, during both the immunohistochemistry for ΔFosB and *in situ* hybridization for GAD65 and TH, we eliminated RNAse and DNAse from the work station and all equipment used by applying an RNAse/DNAse erase decontaminant regularly and intermittently during the experiments. Additionally, sterile labware and certified disposable DNAse/RNAse free materials were used during the experiments. Moreover, diethylpyrocarbonate (DEPC, Sigma-Aldrich) was added to all solutions used for the immunohistochemistry before the *in situ* hybridization was performed.

### Quantification of Immunoreactive Cells

To estimate the number of ΔFosB-positive cells in the various regions of interest, we used the Image-Pro Plus Software version 10.0 (Media Cybernetics, Silver Spring, MD, USA). By comparing each brain section with the corresponding section in the Paxinos rat brain atlas (Paxinos and Watson, 2009), the outlines of regions of interest which are relatively small in size (PVN, VTA, LC, and PBN) were made under the 20× objective of the Olympus BX61 microscope (Olympus Canada, Richmond Hill, ON Canada). For these regions, we analyzed the actual brain sections. For regions of interest that are relatively large (mPFC, Acb, and IC), sections were first scanned using the TISSUEScope 4000 scanner (Huron Digital Pathology, St. Jacobs, ON, Canada) to obtain high-quality images of sections for subsequent analysis. ΔFosB-expressing neuron quantification was performed automatically. To do this, the Image-Pro Plus software was used to identify objects within the regions of interest. Subsequently, the software was fine-tuned continuously by the experimenter until the majority of the objects considered to be neurons in the region of interest were identified by the software. The parameters used were color, area (in pixels: 90–1,500), and size (length: 10–90; width: 5–60). At this point, the value of each parameter was noted and applied to all sections containing regions of interest for analysis. The software was then used to automatically identify all similar objects and the number of objects identified was considered as the number of neurons obtained. To verify the results obtained with the automatic counting, we also performed manual cell counting on some brain sections. The results of both automatic and manual counting were similar. For each brain, the number of neurons identified to express ΔFosB was obtained by averaging the number of ΔFosB-expressing neurons per section in regions of interest in both hemispheres of the brain. The regions of interest were the mPFC [prelimbic (PrL) and infralimbic (IL) cortices; +3.72 mm to +2.72 mm from the bregma], Acb (core and shell; +2.28 mm to +0.96 mm), VTA (−4.80 mm to −5.04 mm), IC (+4.2 mm to +0.12 mm), PBN (medial and lateral parts; −8.88 mm to −9.24 mm), PVN (magnocellular and parvocellular parts; −1.72 mm to −1.92 mm), and LC (−9.60 mm to −9.96 mm).

**Figure 1:**
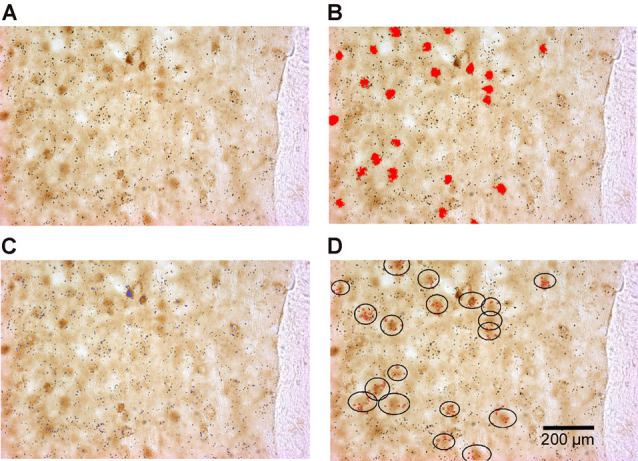
Identification of double-labeled cells (cells expressing both ΔFosB and GAD65 mRNA in this example) using the Image-Pro Plus software and a custom-written MATLAB script. **(A)** An example of a scanned rat brain section showing ΔFosB-expressing cells (dark brown staining) and GAD65 mRNA expression (dark silver grains). **(B)** Identified ΔFosB-expressing cells (red color) using the Image-Pro Plus software. **(C)** Detected GAD65 mRNA expression (blue color) using the Image-Pro Plus software. **(D)** Identified double-labeled cells using a custom-written MATLAB script. In the black circles are ΔFosB-expressing cells that contain more than five dark silver grains (red dots).

To quantify double-labeled cells, all sections were scanned using the TISSUEScope 4000 scanner to obtain high-quality images of sections and the regions of interest (an example of a typical GAD/ΔFosB-labeled section is shown in Figure 1A). ΔFosB-expressing cells were then identified in all outlined regions of interest (Figure 1B), as previously described. The Image-Pro Plus software-defined the coordinates of all identified ΔFosB-expressing cells using the parameters Center X and Center Y. The coordinates of all cells were then exported into Excel files. Similarly, GAD or TH mRNA expression obtained by *in situ* hybridization, which appears as dark silver grains, were also identified based on specific parameters [(in pixels) area: 1–90; size (length): 1–20; size (width): 1–20; Figure 1C] and their coordinates were exported into Excel files. By using a custom-written MATLAB script, double-labeled cells were identified when there was an overlap of ΔFosB expression and mRNA expression at the same location as shown in Figure 1D. The least number of dark silver grains required for a cell to be considered as double-labeled was set to 5. In addition to the number of double-labeled cells, cells expressing ΔFosB only were also identified using the MATLAB script.

### Statistical Analysis

The two-tailed, unpaired student’s *t*-test was used to compare sucrose intake and time spent in the light zone, dark zone, and zone of sucrose between BEP and BER rats during the 10-min modified light/dark box test. Additionally, the two-tailed, unpaired student’s *t*-test was used to compare the difference in means of the number of ΔFosB-expressing and double-labeled (ΔFosB/GAD65 mRNA and ΔFosB/TH mRNA) neurons in the regions of interest in BEP and BER rats. The ordinary two-way analysis of variance (ANOVA) test followed by the Bonferroni *post hoc* test to correct for multiple comparisons was used to compare the total quantities of sucrose solution consumed (dependent variable) by BEP and BER rats (independent variable 1) during sessions with and without foot-shock stress (independent variable 2). The Bonferroni corrected *p*-value was used for the analyses. The interaction between these two independent variables was also assessed. Data are expressed as mean ± standard error of the mean (SEM). Differences in means were considered significant when *p* < 0.05. The statistical tests were performed using GraphPad version 6.01 (GraphPad Software Inc., La Jolla CA, USA).

## Results

### Sucrose Intake During Phenotyping

**Figure 2:**
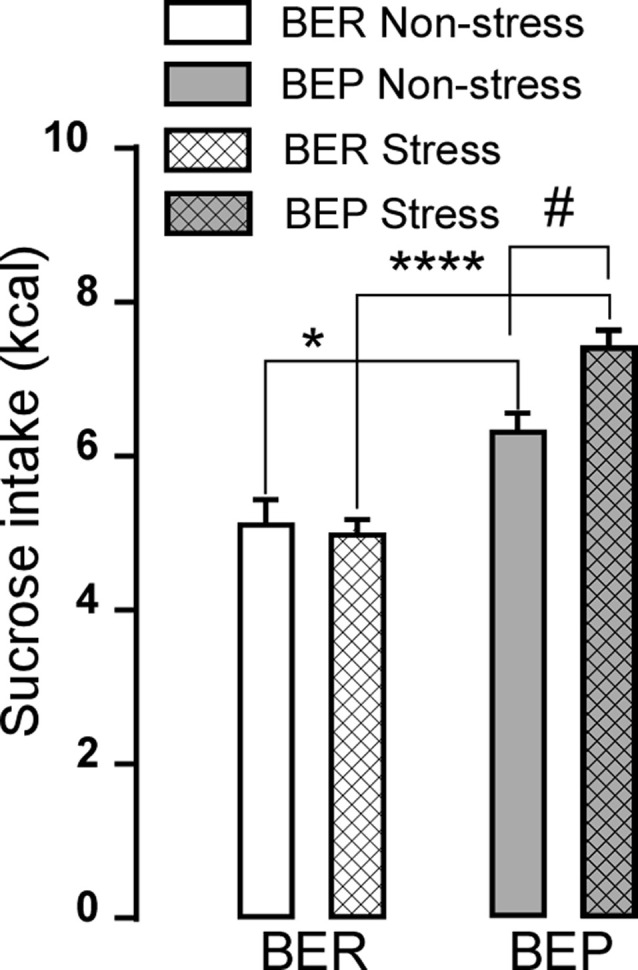
Sucrose intake during phenotyping. The graph shows the 10% sucrose consumption in kilocalories by binge eating prone (BEP) and binge eating resistant (BER) rats during a 1-h access without foot-shock stress (non-stress) and after foot-shock stress (stress). Comparison between phenotypes *p < 0.05; ****p < 0.0001; comparison within phenotype ^#^*p* < 0.05.

Similar to the sucrose intake of BEP and BER rats generated in the study of Calvez and Timofeeva (2016), during 1 h sessions, BER rats consistently consumed smaller amounts of 10% sucrose solution compared to BEP rats both during non-stress and stress sessions (Figure 2). BEP rats increased their intake of sucrose solution after foot-shock stress, while the BER rats consumed similar amounts of sucrose solution both during sessions with and without foot-shock stress (*p* > 0.9999). The effects of phenotype, treatment, and interaction between phenotype and treatment on sucrose consumption in BEP and BER rats were *F*_(1,42)_ = 47.41, *p* < 0.0001; *F*_(1,42)_ = 3.294, *p* = 0.0767; and *F*_(1,42)_ = 5.611, *p* = 0.0225, respectively.

### Sucrose Consumption Under an Aversive Condition in BER and BEP Rats

**Figure 3:**
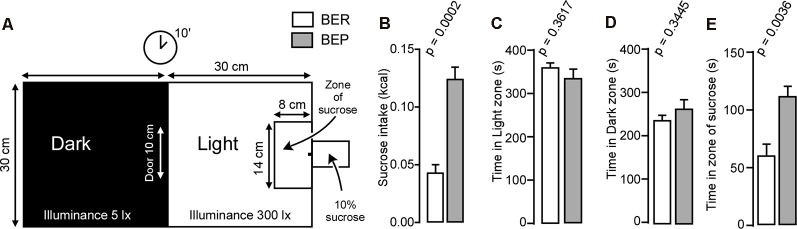
Light/dark box experiment. **(A)** An illustration of the modified light/dark box used during the behavioral experiment. **(B)** Amount of sucrose in kilocalories consumed by BER and BEP rats during the 10-min session in the light/dark box. **(C)** Amount of time in seconds spent by BER and BEP rats in the light zone of the light/dark box. **(D)** Amount of time in seconds spent by BER and BEP rats in the dark zone of the light/dark box. **(E)** Amount of time in seconds spent by BER and BEP rats in the zone of sucrose of the light/dark box. lx: luxes.

We used the modified light/dark box test (Figure 3A) to assess compulsivity in rats. Rats were allowed to explore the box for 10 min with *ad libitum* access to a 10% sucrose solution in the light zone. BEP rats consumed more sucrose than BER rats during the 10-min *ad libitum* access to sucrose in the modified light/dark box (Figure 3B). The zones of interest within the modified light/dark box were the dark zone, light zone, and zone of sucrose. BER and BEP rats spent similar amounts of time in both the light (Figure 3C) and dark (Figure 3D) zones of the box. However, BEP rats spent significantly more time within the zone of sucrose than BER rats (Figure 3E).

### ΔFosB Expression in Reward, Taste, and Stress Systems

The brains of BER and BEP rats were harvested three to four days after the modified light/dark box test was performed. ΔFosB expression was analyzed in different brain regions involved in reward, taste, and stress processing to assess their implication in the development of binge-like eating.

**Figure 4:**
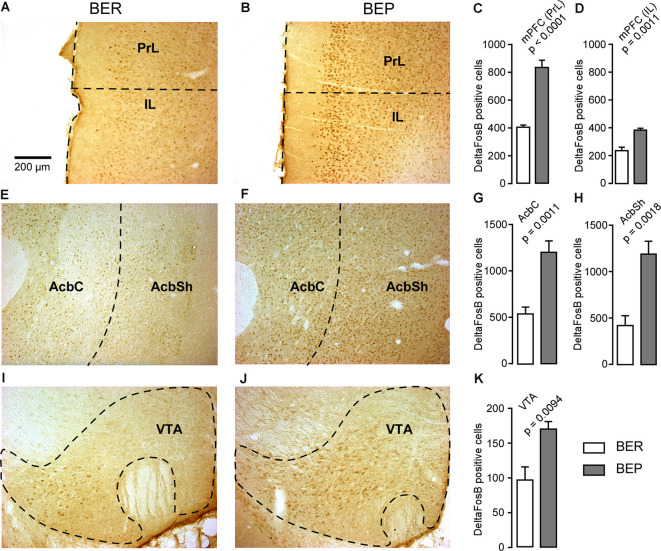
ΔFosB expression in neurons in reward processing regions. **(A,B)** Images showing ΔFosB-expression in neurons in the prelimbic (PrL) and infralimbic (IL) cortices of the medial prefrontal cortex (mPFC) in BER and BEP rats. Inset: a schematic showing the location from which the images in **(A)** and **(B)** were acquired. **(C,D)** The number of ΔFosB-positive cells in the PrL and IL of the mPFC in BEP and BER rats. **(E,F)** Images showing ΔFosB-expression in neurons in the nucleus accumbens core (AcbC) and shell (AcbSh) in BER and BEP rats. **(G,H)** The number of ΔFosB-positive cells in the AcbC and AcbSh in BEP and BER rats. **(I,J)** Images showing ΔFosB-expression in neurons in the ventral tegmental area (VTA) in BEP and BER rats. **(K)** The number of ΔFosB-expressing cells in the VTA of BEP and BER rats. Scale bar: 200 μm.

**Figure 5:**
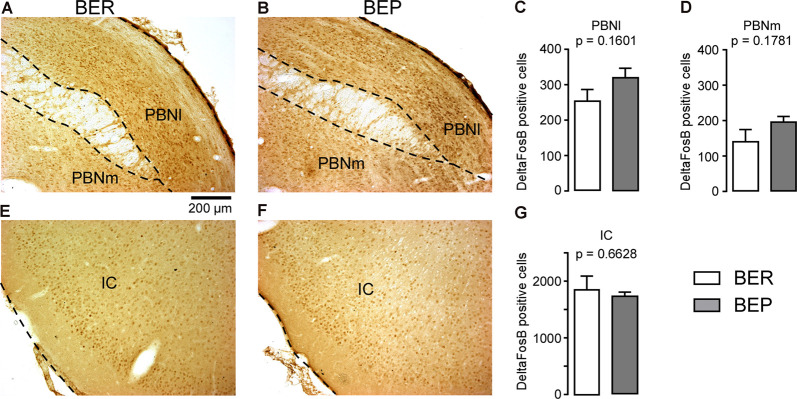
ΔFosB expression in neurons in taste processing regions. **(A,B)** Images showing ΔFosB-expression in neurons in the lateral and medial parts of the parabrachial nucleus (PBN; PBNl and PBMm, respectively) of BEP and BER rats. **(C,D)** The number of ΔFosB-positive cells in the PBNl and PBNm in BEP and BER rats. **(E,F)** Images showing ΔFosB-expression in neurons in the insular cortex (IC) of BEP and BER rats. **(G)** The number of ΔFosB-positive cells in the IC of BEP and BER rats. Scale bar: 200 μm.

**Figure 6:**
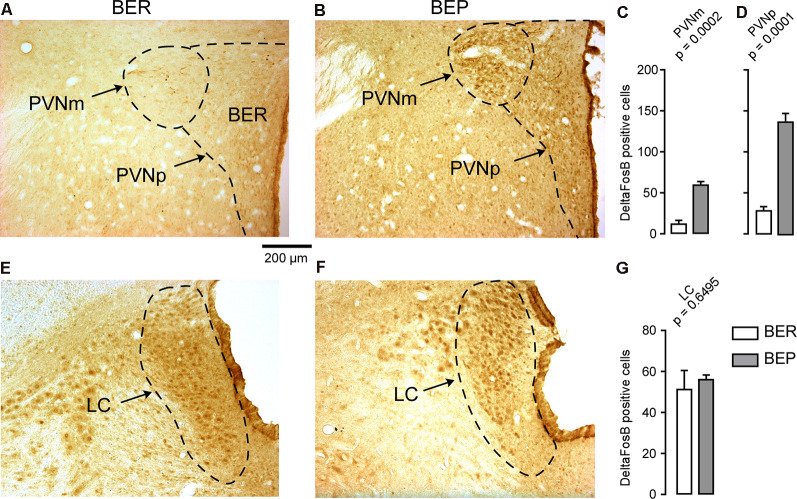
ΔFosB expression in neurons in stress processing regions. **(A,B)** Images showing ΔFosB-expression in neurons in the magnocellular and parvocellular parts of the paraventricular nucleus of the hypothalamus (PVNm and PVNp, respectively) of BEP and BER rats. **(C,D)** The number of ΔFosB-positive cells in the PVNm and PVNp in BEP and BER rats. **(E,F)** Images showing ΔFosB-expression in neurons in the locus coeruleus (LC) of BEP and BER rats. **(G)** The number of ΔFosB-positive cells in the LC of BEP and BER rats. Scale bar: 200 μm.

The number of ΔFosB-expressing cells in all investigated reward processing regions in BEP rats was significantly higher than that in BER rats (Figure 4). A significant difference in the number of ΔFosB-expressing cells was observed in the mPFC, with a higher number of ΔFosB-expressing cells in the PrL and IL of BEP rats compared to BER rats (Figures 4A–D). ΔFosB expression was significantly higher in BEP than in BER rats in the AcbC and AcbSh (Figures 4E–H). There were also more ΔFosB-expressing cells in the VTA of BEP rats compared to BER rats (Figures 4I–K). ΔFosB expression was also analyzed in a subset of taste processing regions including the IC and PBN. Similar numbers of ΔFosB-expressing cells were identified in both the medial and lateral parts of the PBN in BEP and BER rats (Figures 5A–D), as well as in the IC (Figures 5E–G). We also analyzed ΔFosB expression in two stress processing regions: the LC and PVN (Figure 6). Our analyses revealed that there was a significantly higher number of ΔFosB-expressing cells in both the magnocellular and parvocellular parts of the PVN of BEP rats (Figures 6A–D). However, the expression of ΔFosB in the LC of BEP and BER rats (Figures 6E–G) was similar. These results show an increase in ΔFosB expression in reward processing areas and in one of the analyzed stress regions, but not in taste processing areas in BEP rats as compared to BER rats.

### Neuronal Types Implicated in Binge-Like Eating in Reward Processing Regions in the Brain

#### Nucleus Accumbens

**Figure 7:**
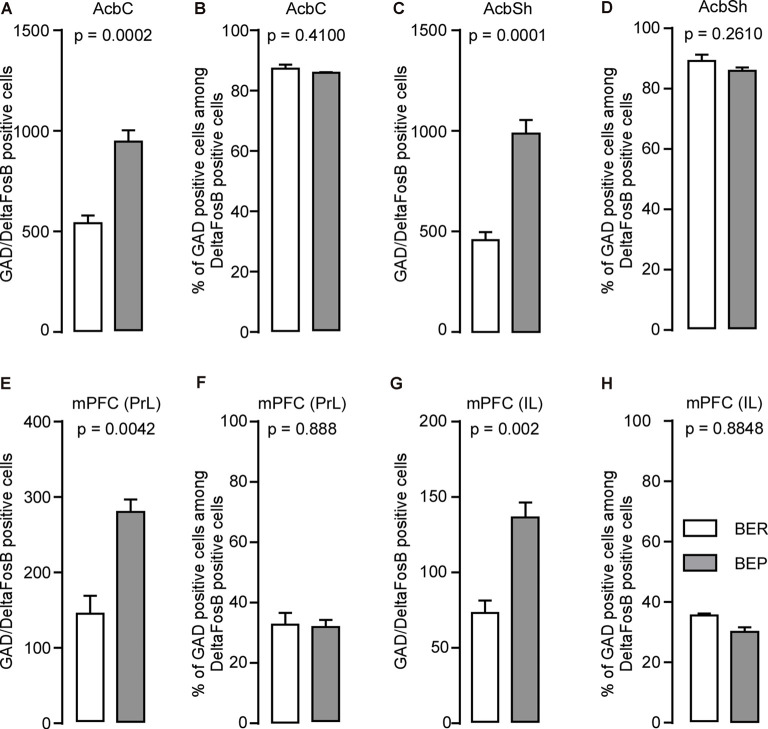
ΔFosB and glutamic acid decarboxylase 65 (GAD65) mRNA expression in neurons in the nucleus accumbens (Acb) and medial prefrontal cortex (mPFC) in BEP and BER rats. **(A,B)** The number and percentage of double-labeled cells (on all ΔFosB cells, percentage that express GAD65 mRNA) in the nucleus accumbens core (AcbC) in BEP and BER rats. **(C,D)** The number and percentage of double-labeled cells in the nucleus accumbens shell (AcbSh) in BEP and BER rats. **(E,F)** The number and percentage of double-labeled cells in the prelimbic cortex (PrL) of the medial prefrontal cortex (mPFC) in BEP and BER rats. **(G,H)** The number and percentage of double-labeled cells in the infralimbic cortex (IL) of the mPFC in BEP and BER rats. The percentages were calculated as follows: (number of GABAergic ΔFosB-expressing cells; total number of ΔFosB-expressing cells) *100.

About 95% of neurons in the Acb are GABAergic cells (Self, 2010). Therefore, we investigated whether the increase in ΔFosB expression during our phenotyping was due to the activation of these cells exclusively or other types of neurons. We found that the number of cells expressing both GAD65 mRNA and ΔFosB was higher in the AcbC (Figure 7A) and AcbSh (Figure 7C) in BEP compared to BER rats. For both phenotypes, the proportion of ΔFosB-expressing cells that also expressed GABA was not significantly different and was 85–90% in the AcbC (Figure 7B) and AcbSh (Figure 7D), suggesting that ΔFosB expression occurred mainly in GABAergic cells but also in non-GABAergic cells in the Acb of both BEP and BER rats.

#### Medial Prefrontal Cortex

About 20% of mPFC neurons are GABAergic, while the remaining are glutamatergic (Gabbott et al., 1997). We found that a higher number of neurons co-expressed ΔFosB and GAD65 mRNA in the PrL of BEP compared to BER rats (Figure 7E). The findings were similar in the IL where more ΔFosB-expressing neurons also expressed GAD65 mRNA in BEP compared to BER rats (Figure 7G). The percentage of ΔFosB-positive neurons that expressed GAD65 mRNA were similar in the PrL (Figure 7F) and IL (Figure 7H) of both BEP and BER rats and was about 30% of the population of the ΔFosB-expressing neurons, suggesting that ΔFosB expression occurred mainly in non-GABAergic cells in the mPFC of both BEP and BER rats.

#### Ventral Tegmental Area

**Figure 8:**
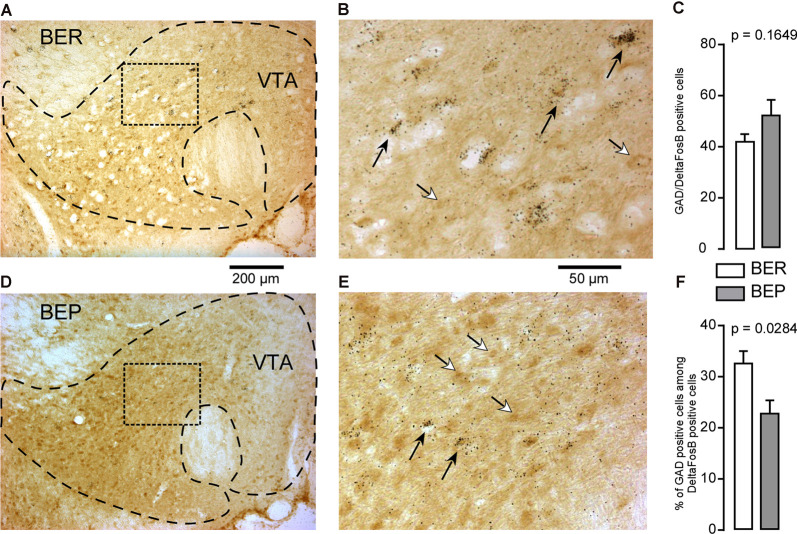
ΔFosB and glutamic acid decarboxylase 65 (GAD65) mRNA expression in neurons in the VTA in BEP and BER rats. **(A,B)** Images showing the labeling of ΔFosB (dark brown) and GAD65 mRNA (dark silver grains) in neurons in the VTA in BER rats. **(C)** The number of ΔFosB/GAD65 mRNA-expressing cells in the VTA in BEP and BER rats. **(D,E)** Images showing the labeling of ΔFosB and GAD65 mRNA in neurons in the VTA in BEP rats. **(F)** The percentage of double-labeled cells (on all ΔFosB cells, the percentage that expresses GAD65 mRNA) in the VTA of BER and BEP rats. Black arrows point to double-labeled neurons, white arrows point to ΔFosB only labeled cells. Scale bar: **(A,C)** = 200 μm, **(B,D)** = 50 μm.

The majority of the neurons in the VTA (65%) are dopaminergic neurons, followed by GABAergic neurons which make up 30%, and then glutamatergic neurons which make up about 5% of the total neuron population (Nair-Roberts et al., 2008). In the VTA, ΔFosB-positive cells which also expressed GAD65 mRNA were observed (Figures 8A,B,D,E). There was no difference in the number of GABAergic ΔFosB-expressing cells identified in BEP and BER rats (Figure 8C). Interestingly, because the total number of ΔFosB-expressing cells was significantly higher in the VTA of BEP compared to BER rats (Figures 4I–K), the percentage of double-labeled cells in the VTA was significantly higher in BER rats compared to BEP rats (Figure 8F).

**Figure 9:**
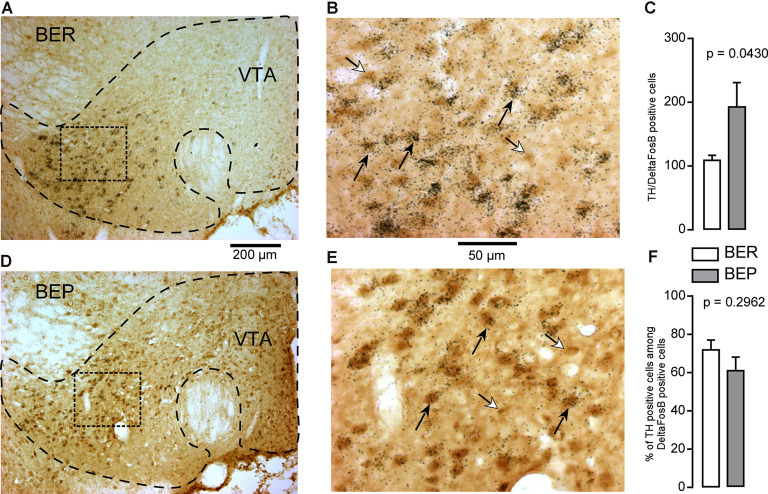
ΔFosB and tyrosine hydroxylase (TH) mRNA expression in neurons in the VTA in BEP and BER rats. **(A,B)** Images showing the labeling of ΔFosB (dark brown) and TH mRNA (dark silver grains) in neurons in the VTA in BER rats. **(C)** The number of ΔFosB/TH mRNA-expressing cells in the VTA in BEP and BER rats. **(D,E)** Images showing the labeling of ΔFosB and TH mRNA in neurons in the VTA in BEP rats. **(F)** The percentage of double-labeled cells (on all ΔFosB cells, the percentage that expresses TH mRNA) in the VTA of BER and BEP rats. Black arrows point to double-labeled neurons, white arrows point to ΔFosB only labeled cells. Scale bar: **(A,C)** = 200 μm, **(B,D)** = 50 μm.

Cells which co-expressed ΔFosB and TH-mRNA were observed in the VTA (Figures 9A,B,D,E). The number of dopaminergic ΔFosB-expressing cells in the VTA of BEP rats was significantly higher than that in BER rats (Figure 9C). However, there was no difference in the percentage of dopaminergic ΔFosB-expressing cells (Figure 9F) in BEP and BER rats.

## Discussion

Binge-like eating rats in this study consumed a large amount of palatable food and bingeing was triggered by stress, which suggests that reward, taste, and stress processing brain regions may be involved (Wolff et al., 2000; American Psychiatric Association, 2013). To verify these hypotheses, we evaluated ΔFosB expression in neurons in these regions. ΔFosB is expressed after repeated neuronal stimulation, and our binge-like eating rat model was developed using repeated accesses to sucrose and several foot-shock stresses. Our results show that the main brain regions implicated in BE are the reward processing regions (mPFC, Acb, and VTA). In BEP rats, the number of ΔFosB-positive neurons was higher in these regions than in BER rats. Additionally, even though the proportions of non-GABAergic and GABAergic neurons in the mPFC, GABAergic neurons in the Acb, and dopaminergic neurons in the VTA were similar in BEP and BER rats, the proportion of VTA ΔFosB-positive neurons that were GABAergic was different between the two phenotypes.

ΔFosB, unlike c-fos which degrades after transient expression, is expressed following chronic stimulation (Wallace et al., 2008; Muñoz-Escobar et al., 2019), which suggests that ΔFosB expression may represent tolerance to persistent stimulation through the reduction of the responsiveness of ΔFosB-expressing neurons to these stimulations (Nestler et al., 1999). As a transcription factor, it regulates the expression of genes including the gene responsible for the expression of the glutamate receptor subunit 2 (GluR2) of the AMPA receptor in Acb neurons (McClung and Nestler, 2003). The upregulation of the GluR2 subunit of the AMPA receptor in ΔFosB-expressing cells introduces an additional positive charge into the AMPA receptor pore, which prevents the passage of divalent cations like Ca^2+^ (Isaac et al., 2007). This, therefore, reduces the permeability of Ca^2+^, thereby reducing the excitability of Acb ΔFosB-expressing neurons. Thus, it has been shown that ΔFosB expression in Acb medium spiny neurons correlated with reduced excitability (Vialou et al., 2010). We observed high ΔFosB expression in the Acb of BEP rats. This finding is similar to that of Muñoz-Escobar et al. (2019) who reported a high expression of ΔFosB in the Acb of bingeing rats. These results suggest that the excitability of Acb medium spiny neurons was significantly reduced (Vialou et al., 2010) in BEP compared to BER rats. Several studies showed that a decrease in neuronal firing in the Acb induced an increase in food consumption whereas stimulation decreased it (Maldonado-Irizarry et al., 1995; Krause et al., 2010; O’Connor et al., 2015). It was also shown that inhibition of the Acb leads to an increase in the response to Olausson et al. (2006) and consumption of Wallace et al. (2008) a reward. Additionally, Acb stimulation in mice alleviates binge eating (Halpern et al., 2013). The high ΔFosB expression in the Acb may be linked to the high sucrose intake observed in our BEP group. This suggests that the reduced Acb activity reported in patients with BE (Balodis et al., 2014) may be associated with high ΔFosB expression in the Acb and may explain the high amounts of palatable food consumed by these patients.

Similar to the Acb (Vialou et al., 2010), ΔFosB expression in the hippocampus also decreases neuronal activity (Eagle et al., 2018). We, therefore, postulate that the activity of ΔFosB-expressing neurons in both the mPFC and VTA would also decrease, and this decrease is greater in BEP than in BER rats. However, further studies are needed to confirm the effects of ΔFosB expression in other brain regions. Our results extend the findings of neuroimaging studies which revealed a reduction in activity in the mPFC (Filbey et al., 2012; Balodis et al., 2013; Reiter et al., 2017), VTA (Bello and Hajnal, 2010; Cordeira et al., 2010), and Acb (Balodis et al., 2014) in patients with BE. Our results are also consistent with the study by Rada et al. (2010) who also reported a decrease in Acb dopamine in bingeing rats, which suggests a decrease in the activity of VTA neurons. Furthermore, a decrease in mPFC activity is associated with compulsivity (Sarica et al., 2018; Sinclair et al., 2019). Patients with BE display compulsive behavior, which is associated with a loss of inhibitory control due to hypoactivity in the mPFC (Balodis et al., 2013; Reiter et al., 2017). Our modified light/dark box test showed that BEP rats spent more time in the zone of sucrose and consumed more sucrose than BER rats. However, both BEP and BER rats spent similar amounts of time in the light zone and spent more time in the light zone than the dark zone. These results suggest that the light intensity used in this test was likely not aversive enough for both groups of rats. However, if the light intensity is too high, rats from both groups will not explore the light zone, and thus not find the sucrose solution. We associate the fact that both BER and BEP rats spent more time in the light zone than the dark zone to the presence of the sucrose solution in the light zone. However, even if both groups spent the same amount of time in the light zone, BEP rats spent more time in the sucrose zone and consumed more sucrose than BER rats in a very short period (10 min). We cannot conclude that BEP rats were more compulsive than BER rats, and other behavioral experiments should be performed to assess the compulsivity of the BEP rats. However, ΔFosB expression in the mPFC of BEP rats was higher than in BER rats, and this region is involved in palatable food consumption and inhibitory control (Killcross and Coutureau, 2003; Aron et al., 2004). Since ΔFosB reduces neuronal excitability in the Acb and the hippocampus (Vialou et al., 2010; Eagle et al., 2018) and mPFC inhibition results in compulsivity (Sinclair et al., 2019), we postulate that a decrease in the mPFC activity may contribute to the binge-eating behavior observed in BEP rats.

Neurons in the taste processing regions (IC and PBN) expressed ΔFosB, and the number of ΔFosB-expressing neurons in these regions was similar in the two phenotypes. BE involves the consumption of highly palatable foods and the IC and PBN are involved in the processing of taste (Norgren and Leonard, 1971; Wise, 2006). The similarity in the number of ΔFosB-expressing neurons in the IC and PBN of BEP and BER rats suggests that both phenotypes processed the palatable food (sucrose) taste similarly, even though BEP rats consumed more sucrose than BER rats, and that these regions may not be directly implicated during BE.

Neurons in the LC, a structure with multiple functions, including stress processing, also expressed ΔFosB even though the number of ΔFosB-expressing neurons was not different between the two phenotypes. Acute stressful stimuli caused an increase in single-unit activity in LC neurons and plasma norepinephrine levels (Abercrombie and Jacobs, 1987). Acute stress activates the LC (Borodovitsyna et al., 2018). We did not find a difference at the level of LC ΔFosB expression in the two investigated groups of animals likely because we used repeated foot-shock stresses in our study, and because the same parameters were applied to both phenotypes. This also suggests that the LC might not be directly involved in BE.

Stress also activates the hypothalamic-pituitary-adrenal (HPA) axis. ΔFosB expression in the PVN was higher in BEP than in BER rats. We showed previously that these BEP rats displayed a blunted stress-induced activation of the HPA axis, with disruption in the levels of corticosterone and corticotropin-releasing factor (CRF; Calvez et al., 2016a). This may be due to the high number of ΔFosB-expressing neurons in the PVN of BEP rats observed after repeated stresses. Our results suggest that the activity of PVN neurons in BEP rats may contribute to BE since stress has been shown to initiate BE in both humans and animals (Levine and Morley, 1981; Willenbring et al., 1986; Epel et al., 2001; Pecoraro et al., 2004; Boggiano et al., 2007) and our rat model is a stress-induced binge eating model.

Our ΔFosB results show that reward processing brain regions are important for developing binge-like eating behavior. Under normal conditions, VTA dopaminergic neurons are activated when a reward is received (Cohen et al., 2012). These dopaminergic neurons subsequently release dopamine in the mPFC (Phillips et al., 2004) and Acb (van Zessen et al., 2012). The released dopamine binds to D1 and D2-receptors on both glutamatergic and GABAergic neurons in the mPFC (Santana et al., 2009) and on GABAergic medium-spiny neurons in the Acb (van Zessen et al., 2012). However, following several stresses (foot-shocks in our experiment), vulnerable rats become binge-eaters (Calvez et al., 2016b). Since ΔFosB expression reduces neuronal activity in the Acb (Vialou et al., 2010) and since more ΔFosB/GABAergic neurons were observed in BEP rats than in BER rats, we conclude that the activity of GABAergic neurons in BEP rats was significantly reduced compared to that in BER rats, and this reduction of inhibitory drive can explain their high sucrose consumption (Krause et al., 2010; Richard et al., 2013b). In the mPFC, there were more GABAergic ΔFosB-expressing neurons in BEP rats than in BER rats. However, as the number of neurons which expressed only ΔFosB was also high in BEP rats, the proportion of ΔFosB/GAD65 mRNA-positive neurons was similar in both phenotypes. We thus conclude that mPFC neuronal activity in BEP rats was significantly reduced compared to that in BER rats, but the proportion of GABAergic and non-GABAergic cells involved are similar for both phenotypes.

In the VTA, the number of dopaminergic ΔFosB-expressing neurons was higher in BEP rats compared to BER rats, but their proportions were similar in the two phenotypes. However, the number of GABAergic ΔFosB-positive neurons was similar in BEP and BER rats. While the expression of ΔFosB in BEP rats was higher, the proportion of GABAergic ΔFosB-expressing neurons was lower in BEP than in BER rats. A difference in the proportion of a neuropeptide in ΔFosB-expressing neurons between the two phenotypes was observed only for the GABAergic neurons in the VTA, which suggests that this region may be the most important structure for the development of binge-like eating. More precisely, it suggests that the GABAergic neuronal activity in the VTA could drive the development of binge-eating. In this region, GABAergic neurons inhibit the activity of dopaminergic neurons. ΔFosB expression is correlated with reduced excitability of ΔFosB-expressing neurons in the Acb and the hippocampus (Vialou et al., 2010; Eagle et al., 2018). As the proportion of GABAergic, ΔFosB-expressing neurons was lower in BEP rats, and if we assume that ΔFosB expression is also correlated with a decrease in excitability in the VTA, it suggests that the GABAergic neuronal activity is less reduced in BEP rats compared to BER rats. In other words, the GABAergic neuronal activity in VTA is higher in BEP rats than in BER rats. This implies an overall decrease in the activity of the other neuron types in the VTA, and of the other structures that receive VTA GABAergic projections in BEP rats, as previously reported in the VTA of patients with BE (Bello and Hajnal, 2010; Cordeira et al., 2010) and in bingeing rats (Rada et al., 2010).

The present study has several limitations that need to be pointed out. The modified light/dark box test was originally performed to assess compulsivity in the rats. However, BER and BEP rats spent similar amounts of time in both the light and dark zones and more time in the light than the dark zones. Thus, a definite conclusion about the compulsivity of rats is not possible. A more robust test for compulsivity should be done, like for example the one described by Oswald et al. (2011), whereby rats were subjected to foot-shock just before access to palatable food. In that case, rats that endure the shock to obtain palatable food can be said to be compulsive. Another limitation is that we did not quantify ΔFosB expression relative to all the different cell populations by using, for example, Nissl or NeuN staining, which would have helped to elucidate the proportions of ΔFosB-expressing cells among the total number of cells in specific regions of the brain. Additionally, one could say that the neuronal differences between BEP and BER rats can be explained by the fact that BEP rats consumed more sucrose than BER rats. The goal of this study was to analyze ΔFosB expression in different brain regions during the development of binge-eating behavior. The characterization of rats as BEP or BER in this study is based on their sucrose consumption after stress. Thus, it was impossible to limit the sucrose access to the rats during the phenotyping, and the higher sucrose consumption of BEP rats is what led to their classification as BEP rats. In future studies, it could be interesting to also analyze the brains of the rats that were not classified as BEP or BER rats (intermediate) to investigate whether ΔFosB expression is similar to that observed in BER and BEP rats or not. Control groups that undergo the same protocol but without the foot-shocks during the stress session could also be analyzed to investigate whether sucrose consumption alone can induce a similar ΔFosB expression. Furthermore, even though we propose that ΔFosB expression may have the same effects in other brain regions as observed in the Acb and the hippocampus, this needs to be evaluated experimentally. Finally, even though we used different methods to maintain the RNA integrity during the immunohistochemistry and *in situ* hybridization, it will be necessary to perform a control *in situ* hybridization without immunohistochemistry and compare the labeling to demonstrate the RNA integrity.

In conclusion, these experiments were designed to analyze, for the first time, ΔFosB expression in different brain regions during the development of binge-like eating in a rat model. We found that the reward system is very important for the development of binge-like eating and that the reduction in activity observed in some animal models of BE and in human studies involving patients with BE in the reward system may be related to the expression of ΔFosB. In this reward system, the proportions of neuron subtypes involved in binge eating were similar in the mPFC and Acb, but different in the VTA in BEP and BER rats. This suggests that these differences in the proportion of ΔFosB-expressing neurons in the VTA may play an important role in binge eating.

## Data Availability Statement

All datasets presented in this study are included in the article/Supplementary Material.

## Ethics Statement

The animal study was reviewed and approved by Université Laval Committee on Ethics and Animal Research.

## Author Contributions

RQA: investigation, formal analysis, visualization, and writing—original draft preparation. SC: project administration, formal analysis, and writing—original draft preparation. JC: investigation, and formal analysis; GG: investigation, project administration, and resources. ET: conceptualization, funding acquisition, methodology, and supervision. IT: formal analysis, funding acquisition, methodology, project administration, supervision, validation, visualization, and writing—review and editing. All authors contributed to the article and approved the submitted version.

## Conflict of Interest

The authors declare that the research was conducted in the absence of any commercial or financial relationships that could be construed as a potential conflict of interest.

## References

[B1] Abercrombie E. D. Jacobs B. L. (1987). Single-unit response of noradrenergic neurons in the locus coeruleus of freely moving cats. I. Acutely presented stressful and nonstressful stimuli. J. Neurosci. 7, 2837–2843. 10.1523/jneurosci.07-09-02837.1987 3625275 PMC6569145

[B2] American Psychiatric Association (2013). Diagnostic and Statistical Manual of Mental Disorders. 5th Edn. Arlington, VA: American Psychiatric Publishing.

[B3] Aron A. R. Robbins T. W. Poldrack R. A. (2004). Inhibition and the right inferior frontal cortex. Trends Cogn. Sci. 8, 170–177. 10.1016/j.tics.2004.02.010 15050513

[B4] Balodis I. M. Grilo C. M. Kober H. Worhunsky P. D. White M. A. Stevens M. C.. (2014). A pilot study linking reduced fronto-Striatal recruitment during reward processing to persistent bingeing following treatment for binge-eating disorder. Int. J. Eat Disord. 47, 376–384. 10.1002/eat.22204 24729034 PMC3986812

[B5] Balodis I. M. Molina N. D. Kober H. Worhunsky P. D. White M. A. Rajita S.. (2013). Divergent neural substrates of inhibitory control in binge eating disorder relative to other manifestations of obesity. Obesity 21, 367–377. 10.1002/oby.20068 23404820 PMC3610836

[B6] Bello N. T. Guarda A. S. Terrillion C. E. Redgrave G. W. Coughlin J. W. Moran T. H. (2009). Repeated binge access to a palatable food alters feeding behavior, hormone profile and hindbrain c-Fos responses to a test meal in adult male rats. Am. J. Physiol. Regul. Integr. Comp. Physiol. 297, R622–R631. 10.1152/ajpregu.00087.2009 19535681 PMC2739780

[B7] Bello N. T. Hajnal A. (2010). Dopamine and binge eating behaviors. Pharmacol. Biochem. Behav. 97, 25–33. 10.1016/j.pbb.2010.04.016 20417658 PMC2977997

[B8] Boggiano M. M. Artiga A. I. Pritchett C. E. Chandler-Laney P. C. Smith M. L. Eldridge A. J. (2007). High intake of palatable food predicts binge-eating independent of susceptibility to obesity: an animal model of lean vs obese binge-eating and obesity with and without binge-eating. Int. J. Obes. 31, 1357–1367. 10.1038/sj.ijo.0803614 17372614

[B9] Borodovitsyna O. Flamini M. D. Chandler D. J. (2018). Acute stress persistently alters locus coeruleus function and anxiety-like behavior in adolescent rats. Neuroscience 373, 7–19. 10.1016/j.neuroscience.2018.01.020 29341884

[B10] Calvez J. de Avila C. Guevremont G. Timofeeva E. (2016a). Stress differentially regulates brain expression of corticotropin-releasing factor in binge-like eating prone and resistant female rats. Appetite 107, 585–595. 10.1016/j.appet.2016.09.010 27616710

[B11] Calvez J. de Avila C. Matte L. O. Guevremont G. Gundlach A. L. Timofeeva E. (2016b). Role of relaxin-3/RXFP3 system in stress-induced binge-like eating in female rats. Neuropharmacology 102, 207–215. 10.1016/j.neuropharm.2015.11.014 26607097

[B12] Calvez J. Timofeeva E. (2016). Behavioral and hormonal responses to stress in binge-like eating prone female rats. Physiol. Behav. 157, 28–38. 10.1016/j.physbeh.2016.01.029 26812591

[B13] Cohen J. Y. Haesler S. Vong L. Lowell B. B. Uchida N. (2012). Neuron-type-specific signals for reward and punishment in the ventral tegmental area. Nature 482, 85–88. 10.1038/nature10754 22258508 PMC3271183

[B14] Cordeira J. W. Frank L. Sena-Esteves M. Pothos E. N. Rios M. (2010). Brain-derived neurotrophic factor regulates hedonic feeding by acting on the mesolimbic dopamine system. J. Neurosci. 30, 2533–2541. 10.1523/JNEUROSCI.5768-09.2010 20164338 PMC2846779

[B15] Corwin R. L. Babbs R. K. (2012). Rodent models of binge eating: are they models of addiction? ILAR J. 53, 23–34. 10.1093/ilar.53.1.23 23520597

[B16] Cunningham J. T. Mifflin S. W. Gould G. G. Frazer A. (2008). Induction of c-Fos and DeltaFosB immunoreactivity in rat brain by Vagal nerve stimulation. Neuropsychopharmacology 33, 1884–1895. 10.1038/sj.npp.1301570 17957222

[B17] Cunningham J. T. Nedungadi T. P. Walch J. D. Nestler E. J. Gottlieb H. B. (2012). DeltaFosB in the supraoptic nucleus contributes to hyponatremia in rats with cirrhosis. Am. J. Physiol. Regul. Integr. Comp. Physiol. 303, R177–R185. 10.1152/ajpregu.00142.2012 22621966 PMC3404636

[B18] Dalley J. W. Everitt B. J. Robbins T. W. (2011). Impulsivity, compulsivity, and top-down cognitive control. Neuron 69, 680–694. 10.1016/j.neuron.2011.01.020 21338879

[B19] Eagle A. L. Williams E. S. Beatty J. A. Cox C. L. Robison A. J. (2018). ΔFosB decreases excitability of dorsal hippocampal CA1 neurons. eNeuro 5:ENEURO.0104-18.2018. 10.1523/ENEURO.0104-18.2018 30079375 PMC6073980

[B20] Epel E. Lapidus R. McEwen B. Brownell K. (2001). Stress may add bite to appetite in women: a laboratory study of stress-induced cortisol and eating behavior. Psychoneuroendocrinology 26, 37–49. 10.1016/s0306-4530(00)00035-4 11070333

[B21] Filbey F. M. Myers U. S. Dewitt S. (2012). Reward circuit function in high BMI individuals with compulsive overeating: similarities with addiction. NeuroImage 63, 1800–1806. 10.1016/j.neuroimage.2012.08.073 22960252

[B22] Friemel C. Spanagel R. Schneider M. (2010). Reward sensitivity for a palatable food reward peaks during pubertal developmental in rats. Front. Behav. Neurosci. 4:39. 10.3389/fnbeh.2010.00039 20700386 PMC2914567

[B23] Gabbott P. L. Dickie B. G. Vaid R. R. Headlam A. J. Bacon S. J. (1997). Local-circuit neurones in the medial prefrontal cortex (areas 25, 32 and 24b) in the rat: morphology and quantitative distribution. J. Comp. Neurol. 377, 465–499. 10.1002/(sici)1096-9861(19970127)377:4<465::aid-cne1>3.0.co;2-0 9007187

[B24] Galmiche M. Déchelotte P. Lambert G. Tavolacci M. P. (2019). Prevalence of eating disorders over the 2000–2018 period: a systematic literature review. Am. J. Clin. Nutr. 109, 1402–1413. 10.1093/ajcn/nqy342 31051507

[B25] Halpern C. H. Tekriwal A. Santollo J. Keating J. G. Wolf J. A. Daniels D.. (2013). Amelioration of binge eating by nucleus accumbens shell deep brain stimulation in mice involves D2 receptor modulation. J. Neurosci. 33, 7122–7129. 10.1523/jneurosci.3237-12.2013 23616522 PMC3703148

[B26] Harrington E. F. Crowther J. H. Henrickson H. C. Mickelson K. D. (2006). The relationships among trauma, stress, ethnicity and binge eating. Cultur. Divers. Ethnic Minor. Psychol. 12, 212–229. 10.1037/1099-9809.12.2.212 16719573

[B27] Herrera D. G. Robertson H. A. (1996). Activation of c-fos in the brain. Prog. Neurobiol. 50, 83–107. 8971979 10.1016/s0301-0082(96)00021-4

[B28] Hetherington M. M. (2000). Eating disorders: diagnosis, etiology and prevention. Nutrition 16, 547–551. 10.1016/s0899-9007(00)00320-8 10906551

[B29] Hudson J. I. Hiripi E. Pope H. G. Jr. Kessler R. C. (2007). The prevalence and correlates of eating disorders in the National Comorbidity Survey Replication. Biol. Psychiatry 61, 348–358. 10.1016/j.biopsych.2006.03.040 16815322 PMC1892232

[B30] Isaac J. T. Ashby M. C. McBain C. J. (2007). The role of the GluR2 subunit in AMPA receptor function and synaptic plasticity. Neuron 54, 859–871. 10.1016/j.neuron.2007.06.001 17582328

[B31] Kaplan M. Jackson B. Sparer R. (1965). Escape behavior under continuous reinforcement as a function of aversive light intensity. J. Exp. Anal. Behav. 8, 321–323. 10.1901/jeab.1965.8-321 14342369 PMC1338103

[B32] Karhunen L. J. Vanninen E. J. Kuikka J. T. Lappalainen R. I. Tiihonen J. Uusitupa M. I. (2000). Regional cerebral blood flow during exposure to food in obese binge eating women. Psychiatry Res. 99, 29–42. 10.1016/s0925-4927(00)00053-6 10891647

[B33] Kessler R. C. Berglund P. A. Chiu W. T. Deitz A. C. Hudson J. I. Shahly V.. (2013). The prevalence and correlates of binge eating disorder in the World Health Organization World Mental Health Surveys. Biol. Psychiatry 73, 904–914. 10.1016/j.biopsych.2012.11.020 23290497 PMC3628997

[B34] Killcross S. Coutureau E. (2003). Coordination of actions and habits in the medial prefrontal cortex of rats. Cereb. Cortex 13, 400–408. 10.1093/cercor/13.4.400 12631569

[B35] Krause M. German P. W. Taha S. A. Fields H. L. (2010). A pause in nucleus accumbens neuron firing is required to initiate and maintain feeding. J. Neurosci. 30, 4746–4756. 10.1523/JNEUROSCI.0197-10.2010 20357125 PMC2878763

[B36] Lee J. E. Namkoong K. Jung Y. C. (2017). Impaired prefrontal cognitive control over interference by food images in binge-eating disorder and bulimia nervosa. Neurosci. Lett. 651, 95–101. 10.1016/j.neulet.2017.04.054 28458022

[B37] Levine A. S. Morley J. E. (1981). Stress-induced eating in rats. Am. J. Physiol. 241, R72–R76. 7195655 10.1152/ajpregu.1981.241.1.R72

[B38] Lundy R. F. Norgren R. (2004). “Gustatory System,” in The Rat Nervous System, ed. Paxinos G. (San Diego, CA and London: Elsevier, Academic Press), 891–921.

[B39] Maldonado-Irizarry C. S. Swanson C. J. Kelley A. E. (1995). Glutamate receptors in the nucleus accumbens shell control feeding behavior via the lateral hypothalamus. J. Neurosci. 15, 6779–6788. 10.1523/jneurosci.15-10-06779.1995 7472436 PMC6578022

[B40] McClung C. A. Nestler E. J. (2003). Regulation of gene expression and cocaine reward by CREB and DeltaFosB. Nat. Neurosci. 6, 1208–1215. 10.1038/nn1143 14566342

[B41] Mitra A. Lenglos C. Martin J. Mbende N. Gagne A. Timofeeva E. (2011). Sucrose modifies c-fos mRNA expression in the brain of rats maintained on feeding schedules. Neuroscience 192, 459–474. 10.1016/j.neuroscience.2011.06.033 21718761

[B42] Muñoz-Escobar G. Guerrero-Vargas N. N. Escobar C. (2019). Random access to palatable food stimulates similar addiction-like responses as a fixed schedule, but only a fixed schedule elicits anticipatory activation. Sci. Rep. 9:18223. 10.1038/s41598-019-54540-0 31796782 PMC6890727

[B43] Nair-Roberts R. G. Chatelain-Badie S. D. Benson E. White-Cooper H. Bolam J. P. Ungless M. A. (2008). Stereological estimates of dopaminergic, GABAergic and glutamatergic neurons in the ventral tegmental area, substantia nigra and retrorubral field in the rat. Neuroscience 152, 1024–1031. 10.1016/j.neuroscience.2008.01.046 18355970 PMC2575227

[B44] Nestler E. J. (2004). Molecular mechanisms of drug addiction. Neuropharmacology 47, 24–32. 10.1016/j.neuropharm.2004.06.031 15464123

[B45] Nestler E. J. Barrot M. Self D. W. (2001). DeltaFosB: a sustained molecular switch for addiction. Proc. Natl. Acad. Sci. U S A 98, 11042–11046. 10.1073/pnas.191352698 11572966 PMC58680

[B46] Nestler E. J. Kelz M. B. Chen J. (1999). DeltaFosB: a molecular mediator of long-term neural and behavioral plasticity. Brain Res. 835, 10–17. 10.1016/s0006-8993(98)01191-3 10448191

[B47] Norgren R. Leonard C. M. (1971). Taste pathways in rat brainstem. Science 173, 1136–1139. 10.1126/science.173.4002.1136 4329178

[B48] O’Connor E. C. Kremer Y. Lefort S. Harada M. Pascoli V. Rohner C.. (2015). Accumbal D1R neurons projecting to lateral hypothalamus authorize feeding. Neuron 88, 553–564. 10.1016/j.neuron.2015.09.038 26593092

[B49] Olausson P. Jentsch J. D. Tronson N. Neve R. L. Nestler E. J. Taylor J. R. (2006). DeltaFosB in the nucleus accumbens regulates food-reinforced instrumental behavior and motivation. J. Neurosci. 26, 9196–9204. 10.1523/JNEUROSCI.1124-06.2006 16957076 PMC6674495

[B50] Oswald K. D. Murdaugh D. L. King V. L. Boggiano M. M. (2011). Motivation for palatable food despite consequences in an animal model of binge eating. Int. J. Eat. Disord. 44, 203–211. 10.1002/eat.20808 20186718 PMC2941549

[B51] Paxinos G. Watson C. (2009). The Rat Brain in Stereotaxic Coordinates. San Diego, CA: Elsevier Academic Press.

[B52] Pecoraro N. Reyes F. Gomez F. Bhargava A. Dallman M. F. (2004). Chronic stress promotes palatable feeding, which reduces signs of stress: feedforward and feedback effects of chronic stress. Endocrinology 145, 3754–3762. 10.1210/en.2004-0305 15142987

[B53] Perrotti L. I. Hadeishi Y. Ulery P. G. Barrot M. Monteggia L. Duman R. S.. (2004). Induction of deltaFosB in reward-related brain structures after chronic stress. J. Neurosci. 24, 10594–10602. 10.1523/jneurosci.2542-04.2004 15564575 PMC6730117

[B54] Perrotti L. I. Weaver R. R. Robison B. Renthal W. Maze I. Yazdani S.. (2008). Distinct patterns of DeltaFosB induction in brain by drugs of abuse. Synapse 62, 358–369. 10.1002/syn.20500 18293355 PMC2667282

[B55] Phillips A. G. Ahn S. Floresco S. B. (2004). Magnitude of dopamine release in medial prefrontal cortex predicts accuracy of memory on a delayed response task. J. Neurosci. 24, 547–553. 10.1523/JNEUROSCI.4653-03.2004 14724255 PMC6729988

[B56] Rada P. Bocarsly M. E. Barson J. R. Hoebel B. G. Leibowitz S. F. (2010). Reduced accumbens dopamine in Sprague-Dawley rats prone to overeating a fat-rich diet. Physiol. Behav. 101, 394–400. 10.1016/j.physbeh.2010.07.005 20643155 PMC2930885

[B57] Reiter A. M. Heinze H. J. Schlagenhauf F. Deserno L. (2017). Impaired flexible reward-based decision-making in binge eating disorder: evidence from computational modeling and functional neuroimaging. Neuropsychopharmacology 42, 628–637. 10.1038/npp.2016.95 27301429 PMC5240187

[B58] Richard J. M. Castro D. C. Difeliceantonio A. G. Robinson M. J. Berridge K. C. (2013a). Mapping brain circuits of reward and motivation: in the footsteps of Ann Kelley. Neurosci. Biobehav. Rev. 37, 1919–1931. 10.1016/j.neubiorev.2012.12.008 23261404 PMC3706488

[B59] Richard J. M. Plawecki A. M. Berridge K. C. (2013b). Nucleus accumbens GABAergic inhibition generates intense eating and fear that resists environmental retuning and needs no dopamine. Eur. J. Neurosci. 37, 1789–1802. 10.1111/ejn.12194 23551138 PMC3672387

[B60] Santana N. Mengod G. Artigas F. (2009). Quantitative analysis of the expression of dopamine D1 and D2 receptors in pyramidal and GABAergic neurons of the rat prefrontal cortex. Cereb. Cortex 19, 849–860. 10.1093/cercor/bhn134 18689859

[B61] Sarica C. Ozkan M. Hacioglu Bay H. Sehirli U. Onat F. Ziyal M. I. (2018). Prelimbic cortex deep brain stimulation reduces binge size in a chronic binge eating rat model. Stereotact Funct. Neurosurg. 96, 33–39. 10.1159/000486965 29533964

[B62] Schafer A. Vaitl D. Schienle A. (2010). Regional grey matter volume abnormalities in bulimia nervosa and binge-eating disorder. NeuroImage 50, 639–643. 10.1016/j.neuroimage.2009.12.063 20035881

[B63] Self D. W. (2010). “Dopamine receptor subtypes in reward and relapse,” in The Dopamine Receptors, ed Neve K. A. (Totowa, NJ: Humana Press), 479–524.

[B64] Sinclair E. B. Culbert K. M. Gradl D. R. Richardson K. A. Klump K. L. Sisk C. L. (2015). Differential mesocorticolimbic responses to palatable food in binge eating prone and binge eating resistant female rats. Physiol. Behav. 152, 249–256. 10.1016/j.physbeh.2015.10.012 26459117 PMC5645798

[B65] Sinclair E. B. Klump K. L. Sisk C. L. (2019). Reduced medial prefrontal control of palatable food consumption is associated with binge eating proneness in female rats. Front. Behav. Neurosci. 13:252. 10.3389/fnbeh.2019.00252 31736726 PMC6834655

[B66] Tanofsky-Kraff M. Bulik C. M. Marcus M. D. Striegel R. H. Wilfley D. E. Wonderlich S. A.. (2013). Binge eating disorder: the next generation of research. Int. J. Eat. Disord. 46, 193–207. 10.1002/eat.22089 23354950 PMC3600071

[B67] van Zessen R. Phillips J. L. Budygin E. A. Stuber G. D. (2012). Activation of VTA GABA neurons disrupts reward consumption. Neuron 73, 1184–1194. 10.1016/j.neuron.2012.02.016 22445345 PMC3314244

[B68] Vialou V. Robison A. J. Laplant Q. C. Covington H. E. Dietz D. M. Ohnishi Y. N.. (2010). DeltaFosB in brain reward circuits mediates resilience to stress and antidepressant responses. Nat. Neurosci. 13, 745–752. 10.1038/nn.2551 20473292 PMC2895556

[B69] Wallace D. L. Vialou V. Rios L. Carle-Florence T. L. Chakravarty S. Kumar A.. (2008). The influence of DeltaFosB in the nucleus accumbens on natural reward-related behavior. J. Neurosci. 28, 10272–10277. 10.1523/JNEUROSCI.1531-08.2008 18842886 PMC2653197

[B70] Willenbring M. L. Levine A. S. Morley J. E. (1986). Stress induced eating and food preference in humans: a pilot study. Int. J. Eat. Disord. 5, 855–864. 10.1002/1098-108x(198607)5:5<855::aid-eat2260050507>3.0.co;2-o

[B71] Wise R. A. (2006). Role of brain dopamine in food reward and reinforcement. Philos. Trans. R. Soc. Lond. B. Biol. Sci. 361, 1149–1158. 10.1098/rstb.2006.1854 16874930 PMC1642703

[B72] Wolff G. E. Crosby R. D. Roberts J. A. Wittrock D. A. (2000). Differences in daily stress, mood, coping, and eating behavior in binge eating and nonbinge eating college women. Addict. Behav. 25, 205–216. 10.1016/s0306-4603(99)00049-0 10795945

[B73] Ziegler D. R. Cass W. A. Herman J. P. (1999). Excitatory influence of the locus coeruleus in hypothalamic-pituitary-adrenocortical axis responses to stress. J. Neuroendocrinol. 11, 361–369. 10.1046/j.1365-2826.1999.00337.x 10320563

