# Supplementary material for: Differential Expression of DeltaFosB in Reward Processing Regions Between Binge Eating Prone and Resistant Female Rats
Source: Front Syst Neurosci. 2020 Oct 16;14:562154. doi: 10.3389/fnsys.2020.562154 (PMC7596303; doi:10.3389/fnsys.2020.562154)
Supplement: Supplementary file 1 [file Table_1.DOCX]

Supplementary Material

# Supplementary Tables

**Data tables showing all data sets used for analyses and for plotting the graphs presented in the paper**

Sucrose intake during phenotyping (kcal)

| Non-stress session | |  | Stress session | |
| --- | --- | --- | --- | --- |
| BER | BEP |  | BER | BEP |
| 4.3 | 5.5 |  | 4.5 | 7.5 |
| 4.0 | 6.2 |  | 3.5 | 7.2 |
| 3.5 | 5.8 |  | 4.4 | 7.1 |
| 5.9 | 5.5 |  | 4.7 | 6.0 |
| 4.0 | 5.4 |  | 4.7 | 6.8 |
| 4.6 | 5.7 |  | 4.6 | 6.7 |
| 5.1 | 8.2 |  | 4.7 | 8.6 |
| 5.5 | 6.9 |  | 6.3 | 8.3 |
| 5.6 | 6.4 |  | 5.2 | 7.7 |
| 7.6 | 6.8 |  | 5.9 | 7.4 |
| 5.0 | 6.9 |  | 5.5 | 8.2 |
| 6.2 |  |  | 5.7 |  |

Light/dark box experiment

| Sucrose  intake (kcal) | |  | Time in dark  zone (s) | |  | Time in light  zone (s) | |  | Time in zone  of sucrose (s) | |
| --- | --- | --- | --- | --- | --- | --- | --- | --- | --- | --- |
| BER | BEP |  | BER | BEP |  | BER | BEP |  | BER | BEP |
| 0.04 | 0.12 |  | 224.2 | 314.4 |  | 371.7 | 284.6 |  | 85.2 | 110.8 |
| 0.06 | 0.09 |  | 194.8 | 220.6 |  | 400.4 | 378.1 |  | 72.0 | 80.1 |
| 0.02 | 0.13 |  | 231.4 | 328.5 |  | 367.7 | 270.8 |  | 41.1 | 138.2 |
| 0.02 | 0.16 |  | 274.3 | 217.7 |  | 325.0 | 381.7 |  | 20.6 | 129.9 |
| 0.07 | 0.14 |  | 277.9 | 205.6 |  | 321.3 | 393.3 |  | 83.7 | 111.4 |
| 0.03 | 0.10 |  | 231.3 | 303.7 |  | 366.8 | 295.6 |  | 55.2 | 96.2 |

Number of deltaFosB-expressing neurons

| mPFC (PrL) | |  | mPFC (IL) | |  | Acb (Core) | |
| --- | --- | --- | --- | --- | --- | --- | --- |
| BER | BEP |  | BER | BEP |  | BER | BEP |
| 385.1 | 840.7 |  | 147.8 | 468.2 |  | 233.3 | 854.0 |
| 420.9 | 812.1 |  | 261.8 | 386.7 |  | 754.5 | 1608.3 |
| 324.0 | 1035.3 |  | 191.5 | 367.7 |  | 475.6 | 874.6 |
| 433.0 | 696.5 |  | 224.0 | 337.8 |  | 585.5 | 1472.4 |
| 426.1 | 917.0 |  | 357.9 | 386.3 |  | 699.4 | 1159.0 |
| 432.1 | 701.7 |  | 200.2 | 377.6 |  | 478.5 | 1251.8 |

| Acb (Shell) | |  | VTA | |  | Insula | |
| --- | --- | --- | --- | --- | --- | --- | --- |
| BER | BEP |  | BER | BEP |  | BER | BEP |
| 192.0 | 758.3 |  | 69.1 | 189.4 |  | 2237.3 | 1736.9 |
| 946.8 | 1499.3 |  | 191.1 | 145.9 |  | 2682.9 | 1735.9 |
| 361.4 | 1130.6 |  | 57.3 | 167.4 |  | 1442.4 | 1559.5 |
| 335.4 | 1613.6 |  | 87.8 | 207.4 |  | 1076.8 | 1522.6 |
| 368.4 | 1356.2 |  | 89.9 | 177.1 |  | 2118.1 | 1774.5 |
| 369.0 | 797.3 |  | 82.9 | 129.4 |  | 1517.7 | 2054.9 |

| PVN (Magno) | |  | PVN (Parvo) | |  | PBN (Medial) | |
| --- | --- | --- | --- | --- | --- | --- | --- |
| BER | BEP |  | BER | BEP |  | BER | BEP |
| 6.5 | 64.8 |  | 10.5 | 167.5 |  | 247.5 | 227.5 |
| 16.8 | 68.2 |  | 38.2 | 112.2 |  | 71.0 | 213.2 |
| 22.2 | 67.0 |  | 28.3 | 105.8 |  | 67.0 | 228.8 |
| 0.5 | 45.5 |  | 32.2 | 141.7 |  | 108.5 | 165.2 |
|  | 50.3 |  |  | 149.5 |  | 199.8 | 210.8 |

| PBN (Lateral) | |  | LC | |
| --- | --- | --- | --- | --- |
| BER | BEP |  | BER | BEP |
| 322.5 | 380.5 |  | 49.3 | 50.0 |
| 197.0 | 298.5 |  | 63.3 | 57.0 |
| 341.0 | 384.2 |  | 45.8 | 50.3 |
| 166.5 | 215.9 |  | 11.8 | 61.4 |
| 234.2 | 360.3 |  | 82.0 | 50.5 |
|  | 273.5 |  | 54.2 | 64.8 |

Number of DeltaFosB/GAD65-expressing neurons

| mPFC (PrL) | |  | mPFC (IL) | |  | Acb (Core) | |
| --- | --- | --- | --- | --- | --- | --- | --- |
| BER | BEP |  | BER | BEP |  | BER | BEP |
| 95.0 | 291.4 |  | 61.0 | 108.0 |  | 445.5 | 919.7 |
| 109.2 | 285.5 |  | 68.7 | 148.9 |  | 415.5 | 775.0 |
| 158.5 | 310.8 |  | 87.5 | 154.3 |  | 601.3 | 1149.9 |
| 116.5 | 231.8 |  | 51.5 | 132.8 |  | 603.3 | 916.3 |
| 237.5 |  |  | 96.5 |  |  | 646.5 | 952.8 |
|  |  |  |  |  |  | 534.5 |  |

| Acb (Shell) | |  | VTA | |
| --- | --- | --- | --- | --- |
| BER | BEP |  | BER | BEP |
| 420.5 | 1059.2 |  | 37.5 | 70.5 |
| 529.3 | 1037.8 |  | 38.5 | 49.2 |
| 462.8 | 745.1 |  | 47.0 | 42.3 |
| 316.3 | 1170.3 |  | 51.0 | 46.3 |
| 340.5 | 894.0 |  | 36.3 |  |
| 626.8 |  |  |  |  |

Percentage of DeltaFosB/GAD65-expressing neurons

| mPFC (PrL) | |  | mPFC (IL) | |  | Acb (Core) | |
| --- | --- | --- | --- | --- | --- | --- | --- |
| BER | BEP |  | BER | BEP |  | BER | BEP |
| 41.8 | 30.8 |  | 34.4 | 25.4 |  | 83.3 | 84.9 |
| 25.8 | 29.6 |  | 37.9 | 33.2 |  | 91.0 | 85.2 |
| 36.8 | 27.8 |  | 33.1 | 31.4 |  | 82.7 | 83.6 |
| 20.2 | 39.0 |  | 35.3 | 30.0 |  | 87.6 | 87.1 |
| 38.1 |  |  |  |  |  | 92.0 | 86.8 |
|  |  |  |  |  |  | 86.0 |  |

| Acb (Shell) | |  | VTA | |
| --- | --- | --- | --- | --- |
| BER | BEP |  | BER | BEP |
| 87.7 | 88.3 |  | 40.0 | 30.7 |
| 79.7 | 88.2 |  | 34.2 | 16.8 |
| 89.3 | 86.4 |  | 28.9 | 20.4 |
| 96.6 | 84.5 |  | 34.2 | 18.4 |
| 89.4 | 81.2 |  | 25.3 | 27.2 |
| 91.6 |  |  |  |  |

Number of deltaFosB/TH-expressing neurons

| VTA | |
| --- | --- |
| BER | BEP |
| 73.5 | 159.7 |
| 103.3 | 262.2 |
| 112.8 | 250.0 |
| 127.9 | 88.5 |
| 101.0 |  |

Percentage of deltaFosB/TH-expressing neurons

| VTA | |
| --- | --- |
| BER | BEP |
| 47.3 | 57.3 |
| 76.2 | 81.7 |
| 65.6 | 55.8 |
| 72.9 | 49.2 |
| 90.2 |  |
| 75.3 |  |

Legend

mPFC: medial prefrontal cortex; mPFC (PrL): prelimbic cortex; mPFC (IL): infralimbic cortex, Acb: nucleus accumbens; Acb (Core): nucleus accumbens core; Acb (Shell): nucleus accumbens shell; VTA: ventral tegmental area; PVN: paraventricular nucleus of the hypothalamus; PVN (Magno): magnocellular part of the paraventricular nucleus of the hypothalamus; PVN (Parvo): parvocellular part of the paraventricular nucleus of the hypothalamus; PBN: parabrachial nucleus; PBN (Medial): medial part of the parabrachial nucleus; PBN (Lateral): lateral part of the parabrachial nucleus; LC, locus coeruleus; Insula: insular cortex; BER: binge eating resistant rats; BEP: binge eating prone rats
